# The association between geospatial and temporal factors and pre-hospital response to major trauma: a retrospective cohort study in the North of England

**DOI:** 10.1186/s13049-023-01166-x

**Published:** 2023-12-19

**Authors:** Ryan D McHenry, Christopher A Smith

**Affiliations:** 1 ScotSTAR, Scottish Ambulance Service, Hangar B, 180 Abbotsinch Road, Paisley, PA3 2RY UK; 2 Great North Air Ambulance Service, The Imperial Centre, Darlington, UK

**Keywords:** Pre-hospital emergency medicine, Geospatial modelling, Geographic isolation, Tasking

## Abstract

**Background:**

Major trauma is a leading cause of premature death and disability worldwide, and many healthcare systems seek to improve outcomes following severe injury with provision of pre-hospital critical care. Much research has focussed on the efficacy of pre-hospital critical care and advanced pre-hospital interventions, but less is known about how the structure of pre-hospital critical care services may influence response to major trauma. This study assessed the association between likelihood of pre-hospital critical care response in major trauma and factors important in the planning and development of those services: geographic isolation, time of day, and tasking mechanism.

**Methods:**

A local trauma registry, supported with data from the Trauma Audit and Research Network alongside additional information regarding pre-hospital management, identified patients sustaining major trauma admitted to Major Trauma Centres in the North of England. Data was extracted on location and time of incident, mechanism of injury, on-scene times, and presence or absence of pre-hospital critical care team. An isochrone map was constructed for 30-minute intervals to regional Major Trauma Centres, defining geographic isolation. Univariate logistic regression compared likelihood of pre-hospital critical care response to that of conventional ambulance response for varying degrees of geographic isolation, day or night period, and mechanism of injury, and multiple linear regression assessed the association between geographic isolation, service response and on-scene time.

**Results:**

2619 incidents were included, with 23.3% attended by pre-hospital critical care teams. Compared to conventional ambulance services, pre-hospital critical care teams were more likely to respond major trauma in areas of greater geographic isolation (OR 1.42, 95% CI 1.30–1.55, *p* < 0.005). There were significant differences in the mechanism of injury attended and no significant difference in response by day or night period. Pre-hospital critical care team response and increasing geographic isolation was associated with longer on-scene times (*p* < 0.005).

**Conclusion:**

Pre-hospital critical care teams are more likely to respond to major trauma in areas of greater geographic isolation. Enhanced pre-hospital care may mitigate geographic inequalities when providing advanced interventions and transport of severely injured patients. There may be an unmet need for pre-hospital critical care response in areas close to major hospitals.

**Supplementary Information:**

The online version contains supplementary material available at 10.1186/s13049-023-01166-x.

## Background

Major trauma is a leading cause of premature death and disability worldwide [1, 2]. Advanced pre-hospital care in severe injury has the potential to improve outcomes, and pre-hospital critical care (PHCC) is now well-established within many trauma networks internationally [3, 4]. These services complement conventional ambulance response by providing enhanced interventions, treatment, and decision-making in the pre-hospital phase of care [5]. As PHCC teams are resource-intensive [6], judicious use of PHCC is important within a trauma system. A recent priority-setting partnership for PHCC has identified important questions regarding service configuration, including in the improvement of PHCC dispatch and comparisons of that of conventional ambulance services [7].

Geographic isolation has long been known to pose challenges for the delivery of healthcare, and particularly in the delivery of time-critical interventions in major trauma [8, 9], though the relationship of distance from care and health-related outcomes remains uncertain, with many studies reporting conflicting results [10, 11]. Additionally, the interaction between geographic isolation and availability of advanced pre-hospital intervention, with the potential to stabilise critically ill patients during transport, remains complex and incompletely understood [12]. Various markers of geographic isolation have been considered in efforts to define isolation from care [11]. Isochrone mapping, defining the area accessible to a specific point within a time threshold, respects local geography and transport infrastructure and thus has greater applicability to real-world travel times than simple straight-line distance and has seen use in healthcare service delivery, notably in ambulance service bypass criteria for major trauma centres [13, 14].

The Great North Air Ambulance Service (GNAAS) commenced operations in 1994, covering mixed urban and rural areas in the North of England from bases in Langwathby, Cumbria and Urlay Nook, County Durham. The service operates a dual critical care paramedic and physician model, operating with both rotary-wing and road transport platforms. The model of care delivers PHCC, with a particular target response to major trauma, working alongside and augmenting the work of conventional ambulances services, principally North West Ambulance Service NHS Trust and North East Ambulance Service NHS Foundation Trust. GNAAS tasking is by dedicated non-clinical dispatch during daytime hours, and ambulance service dispatch at night; both typically dispatch the service as a primary response before the arrival of other clinical resources. Response times between tasking and mobilisation are targeted to less than 10 min. Over the study period, GNAAS expanded hours of operations by both air and road. Initially operating between the hours of 0800 and 2000, and from November 2018, by both road and air in daytime hours and by road overnight, with immediate response on Friday and Saturday nights, and on-call response for major incident across the remaining week. Further expansion in June 2022 now brings the service to continuous operations in day and night periods throughout the week.

While many studies have assessed the patient characteristics associated with major trauma and its outcomes, this work aims to expand knowledge of geospatial and temporal factors associated with major trauma, to derive organisational- and service-level factors that affect the dispatch of either a PHCC team or conventional ambulance resource to this patient group. Evidence of these factors has the potential to influence the structure, dispatch and location of PHCC teams, to rationalise delivery of resource-intensive services, and ensure timely attendance and transport following severe injury.

## Methods

The study is reported using the Strengthening the Reporting of Observational Studies in Epidemiology (STROBE) guidelines [15], with a checklist available as Supplementary Table 1.

As in previous reports of pre-hospital response in the region [16], the population was derived from the Trauma Audit Research Network (TARN) dataset for all patients, across all age groups, presenting to hospitals in the North East Ambulance Service (NEAS) area; including all trauma units and the area’s two Major Trauma Centres (MTCs). Patients were included in the analysis if they were a major trauma patient (Injury Severity Score (ISS) ≥ 9) registered in the TARN database and having at least one of the following:

Intubated in the prehospital phase or in the ED.

Received blood products prehospital or in the ED.

Admitted direct to level 2 or 3 critical care from the ED.

Underwent surgery within 4 h of ED arrival.

Hypotensive on arrival to the ED (systolic blood pressure ≤ 90mmHg for all age groups).

Patients were excluded if they:

Did not trigger the prehospital major trauma triage tool.

Self-presented to hospital.

Sustained an isolated neck of femur fracture or a single rib fracture.

Were diagnosed as being in traumatic cardiac arrest and those not conveyed to hospital (as this information is not collected by the TARN dataset).

The population under review included all patients meeting the inclusion criteria from 1st January 2013-31st December 2022 inclusive. On-scene time was derived from pre-hospital critical care and ambulance service electronic records, and reflects the time from scene arrival to scene departure for the specified service. Entries without incident location data were excluded from review.

Isochrones were constructed for modelled travel time by road at 30-minute intervals to the English Northern Trauma Network Major Trauma Centres (MTCs), which receive the majority of patients experiencing severe injury in the region. The sites of major trauma were identified by postcode of incident, and locations were plotted by identifying the centroid of each postcode area. Where full postcode data were not available, the centroid of the postcode district of the incident was used.

Incidents were stratified by modelled time to major trauma centre, time-of-day and day-of-week, and pre-hospital resource attending (dichotomised as conventional ambulance response only, or presence of PHCC team with or without conventional ambulance response).

The time-of-day and day-of-week of each incident was visualised using a heat map, and the further stratified into day and night periods (0800-2000 h and 2000 h-0800 h respectively) for analysis. Incident mechanism was categorised as falls of less than 2 m, falls greater than 2 m, interpersonal violence, vehicle incidents, combined blast, burn or crush, or other mechanism.

Categorical data was reported as frequency and percentages, and continuous data as median and inter-quartile range (IQR). The primary outcome under consideration was the attendance of PHCC team. Univariate logistic regression was used to assess the association between the presence or absence of PHCC team and modelled time to MTC of each incident, day or night period, and mechanism as odds ratios (OR) with 95% confidence intervals (CIs). For analysis, successive isochrones were treated as a continuous variable, and both day or night period and mechanism of injury were treated as categorical variables. Multiple linear regression was used to assess if on-scene time was predicted by the service-level considerations of geographic isolation and response type. Linearity between scene time and ordinal successive isochrones was assessed graphically with box plots (Supplementary Fig. 1). All analysis was of complete-cases for each of the variable pairings, with no imputation of missing data.

Analysis and geospatial modelling was conducted using the sf and osrm packages in R (R Foundation for Statistical Computing, Vienna, Austria) [17, 18].

As observational analysis of routinely-collected data, the project met the definition of service evaluation and did not require formal ethics approval [19].

## Results

Of 4418 incidents included in the dataset, 2619 (59.2%) included incident location data for analysis; 609 (23.3%) attended by PHCC team, and 2010 (76.7%) received conventional ambulance response without PHCC. Cases excluded due to incomplete incident location data comprised of 910 with PHCC team attendance, and 1057 with conventional ambulance response (representing 59.9% and 34.5% of the response from each service respectively).

Table 1 details numbers of complete cases, and their attributes. Case numbers for, and odds ratio of, PHCC response increased for each year; OR 1.10 (95% CI 1.06–1.13).

**Table 1:** Results of univariate logistic regression for associations between attendance of Pre-Hospital Critical Care team and year, modelled time to Major Trauma Centre, day or night period, and mechanism of injury

	Pre-Hospital Critical Care Response	Conventional Ambulance Response	Univariate Odds Ratio for PHCC Response (95% Confidence Interval)	*p* value
	Number of incidents (% of each service type)			
**Year**				
2013	28 (4.6%)	205 (10.2%)	For each year; 1.10 (1.06–1.13)	< 0.005
2014	34 (5.6%)	191 (9.5%)		
2015	54 (8.9%)	216 (10.7%)		
2016	83 (13.6%)	225 (11.2%)		
2017	72 (11.8%)	170 (8.5%)		
2018	45 (7.4%)	185 (9.2%)		
2019	51 (8.4%)	231 (11.5%)		
2020	87 (14.3%)	247 (12.3%)		
2021	67 (11.0%)	149 (7.4%)		
2022	88 (14.4%)	191 (9.5%)		
Total	609 (100%)	2010 (100%)		
**Modelled time to Major Trauma Centre**				
≤ 30 min	292 (47.9%)	1385 (68.9%)	Reference	
31–60 min	182 (29.9%)	394 (19.6%)	For each successive 30-minute isochrone; 1.42 (1.30–1.55)	< 0.005
61–90 min	85 (14.0%)	137 (6.8%)		
91–120 min	30 (4.9%)	42 (2.1%)		
121–150 min	20 (3.3%)	52 (2.6%)		
Total	609 (100%)	2010 (100%)		
**Day or Night Period**				
Day (0800–2000)	427 (82.1%)	1198 (78.9%)	Reference	
Night (2000 − 0800)	93 (17.9%)	321 (21.1%)	0.813 (0.63–1.05)	0.112
Total	520 (100%)	1519 (100%)		
Missing	89	491		
**Mechanism**				
Falls of < 2 m	54 (8.9%)	589 (30%)	Reference	
Falls of > 2 m	135 (22.2%)	495 (24%)	2.97 (2.13–4.20)	< 0.005
Burn, blast or crush	16 (2.6%)	19 (1%)	9.19 (4.43–18.90)	< 0.005
Interpersonal Violence	81 (13.3%)	240 (12%)	3.68 (2.54–5.38)	< 0.005
Vehicle incidents	285 (46.8%)	527 (26%)	5.90 (4.34–8.14)	< 0.005
Other	38 (6.2%)	140 (7%)	2.96 (1.87–4.65)	< 0.005
Total	609 (100%)	2010 (100%)		

**Fig. 1:**
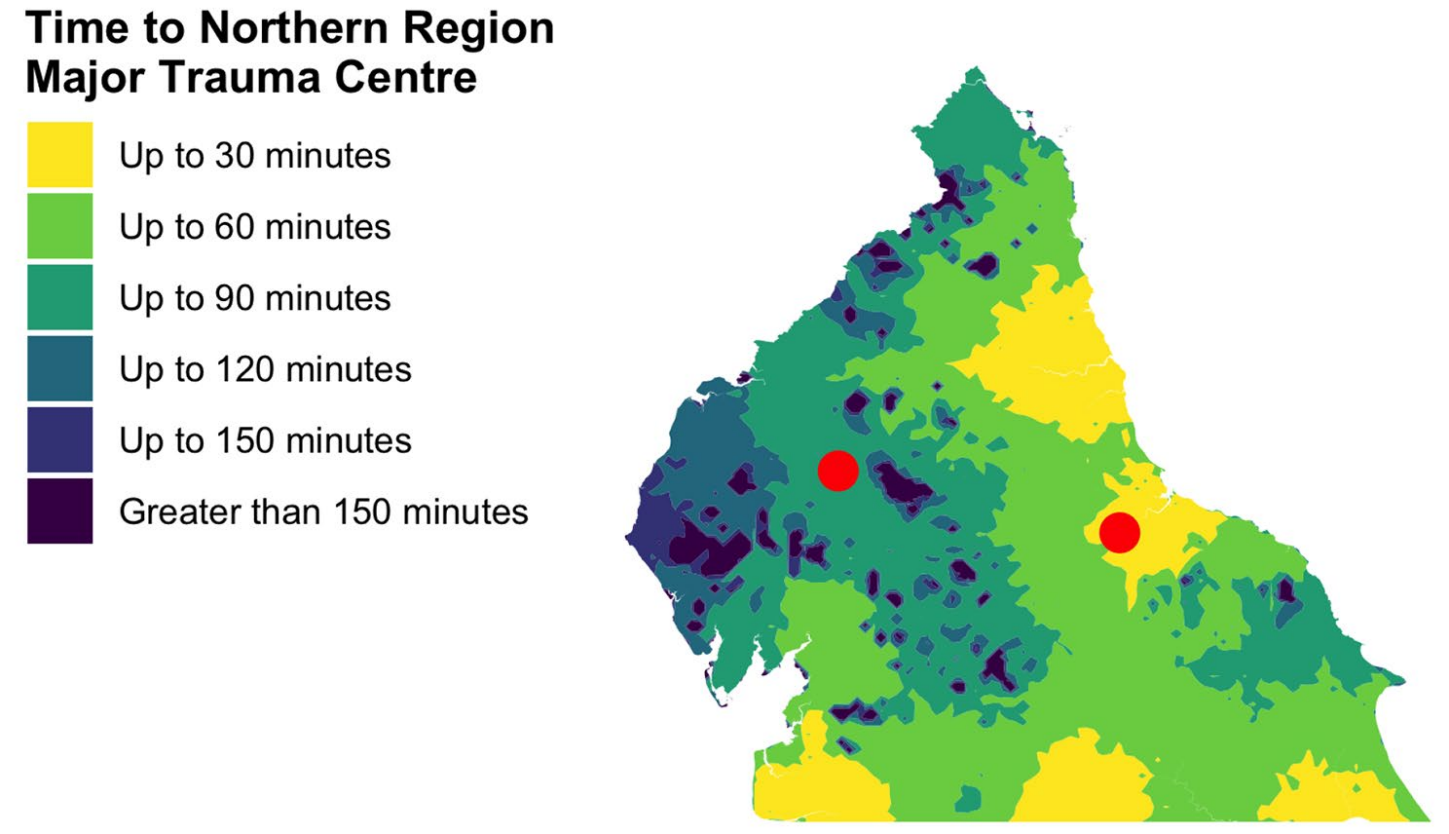
Isochrone map of modelled time to major trauma centres in Northern England

Figure 1 demonstrates the isochrone map of modelled time to each of the regional MTCs.

**Fig. 2:**
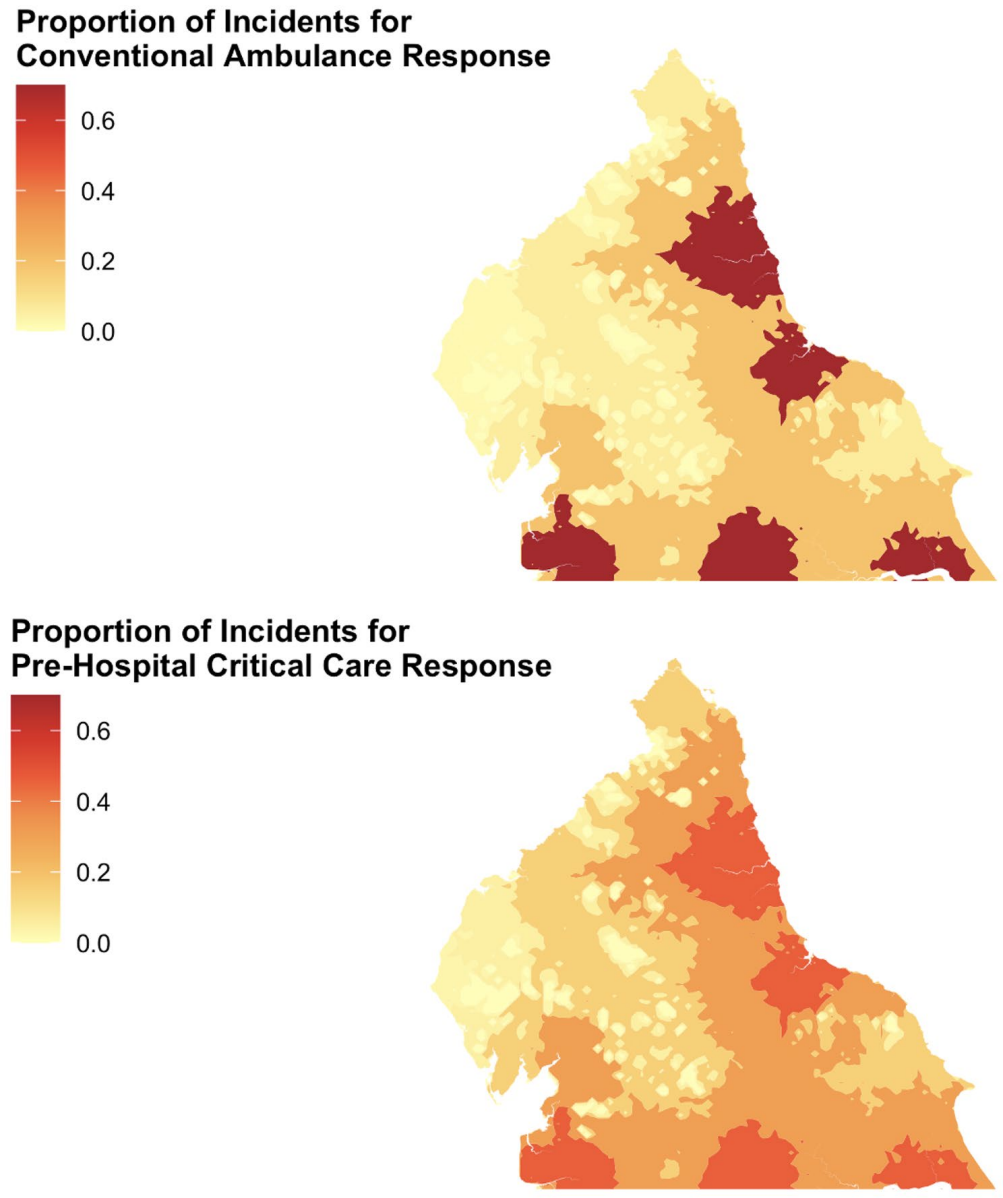
Mapping demonstrating the proportion of each services’ response at each 30 min isochrone

In the study population, 1677 incidents (64.0%) occurred at areas modelled at ≤ 30 min by road to MTC; with fewer incidents at each successive modelled interval of time to MTC. Figure 2 shows the proportion of incidents attended by each service at each 30-minute isochrone. While conventional ambulance resource attended more incidents overall and at each degree of isolation, PHCC teams attended a greater proportion of their workload at greater isolation from MTC than conventional ambulance response.

Compared to the attending service at ≤ 30 min from an MTC, patients were significantly more likely to receive a PHCC response at areas of greater isolation from MTC; OR for PHCC response at each successive minute isochrone from MTC 1.42 (95% CI 1.30–1.55), *p* < 0.005.

There was no significant association between incidents occurring in the day or night period and attendance of PHCC team.

Using falls of < 2 m as a reference, PHCC teams were significantly more likely to attend incidents with a mechanism of greater than 2 m, interpersonal violence, vehicle incidents, burn, blast or crush, and other categories. Table 1 shows results of these univariate logistic regressions. Post-hoc ordinal regression demonstrated association between mechanism and isolation, with vehicle incidents significantly more likely at areas further from MTC (Supplementary Table 1).

Analysis of the service-level predictors of on-scene time with univariate linear regression demonstrated significant differences in on-scene times between PHCC (median 43 min; IQR 31–57 min) and conventional ambulance services (median 34 min; IQR 25–47 min), with HEMS response associated with estimated 7.9 min longer on-scene, *p* < 0.005. On-scene time was also longer at incidents of greater geographic isolation, with estimated increase of 4.4 min for each successive 30-minute isolation from MTC, *p* < 0.005. Both remained significant in multiple linear regression for the effect of isolation and service type on time on-scene, albeit accounting for little of the variance demonstrated; R^2^ = 0.053, *p* < 0.005. Associations between on-scene times, geographic isolation, and pre-hospital response are demonstrated in Table 2.

**Table 2:** Results of linear regression for associations between on-scene time, attendance of Pre-Hospital Critical Care team and modelled time to Major Trauma Centre

	Univariate linear regression		Multiple linear regression	
	Estimate in minutes (95% Confidence Interval)	*p* value	Estimate in minutes (95% Confidence Interval)	*p* value
Geographic Isolation	4.41 (3.37–5.45)	< 0.005	3.79 (2.73–4.84)	< 0.005
Pre-Hospital Critical Care Present	7.78 (5.74–10.02)	< 0.005	6.33 (4.17–8.48)	< 0.005

Geographic isolation effect for each successive 30-minute isochrone from Major Trauma Centre

Variation explained, R^2^ = 0.053 for the multiple linear regression model

## Discussion

Increasing isolation from major trauma centre was associated with greater likelihood of PHCC response, suggesting that the advanced interventions available to PHCC teams have a role in mitigating geographic inequalities experienced by those suffering severe injury distant from definitive care. This work details the geographic spread of major trauma in a single English region, and reflects other literature identifying that the most severe injury occurs at sites close to major trauma centres [20]. The increasing likelihood of PHCC response at areas of geographic isolation is likely to predominantly reflect tasking practices; these may be driven both by the availability to the PHCC team of rotary wing transport, shortening journey times to areas of greater isolation, and of an appreciation that some of the interventions delivered by PHCC may bring more benefit to patients requiring longer transport to definitive care, compared to those who may be quickly conveyed to nearby MTCs. While there is concern that longer pre-hospital times associated with PHCC may delay definitive care, evidence suggests advanced pre-hospital care improves outcomes [3, 4]; combined with this evidence that patients experiencing injury near to MTC are relatively underserved by PHCC, future planning of resource configuration may seek to place advanced care teams closer to these sites. Equally, research may focus on the potential benefits that PHCC may bring specifically to those injured at greater geographic isolation, and seek to resource services to meet these needs.

Spatio-temporal analysis, such as that presented in this work, has the potential to influence service delivery through recognition of associations between geographic factors and response to major trauma, supporting optimal placement of enhanced care teams and describing patterns of workload across time-of-day. These techniques also allow visualisation of the spatio-temporal distribution of incidents, facilitating communication of the importance of these factors to policy-makers, such as through an online app created during the course of this work (available at https://mappingemergencycare.shinyapps.io/NewWorkMap/).

As tasking is key to ensuring timely dispatch of appropriate resource, this work examined the association between mechanism of injury and likelihood of PHCC attendance. While falls of less than 2 m accounted for 30% of the severe injury encountered by ambulance response, advanced care teams were less likely to respond to these incidents, which comprised only 9% of PHCC attendances. Recent work has identified the burden of injury from low falls, particularly in the elderly, and indicates that MTC care improves outcomes for those most severely injured [21], like those included in the present study population. Our work identifies that those with severe injury following a low fall are currently underserved by PHCC resource, and strengthens calls to optimise identification of major trauma and improve PHCC tasking for this group [7, 22].

An association was demonstrated between degree of geographic isolation and injury mechanism, with high falls and vehicle incidents more likely at greater isolation. While this finding is expected in the context of a higher likelihood of serious vehicle incidents on faster, rural roads [23], if vehicle incidents or high falls preferentially precipitate tasking of PHCC, this may help to explain the findings that PHCC has a greater likelihood of attending more isolated incidents. Future work should seek to determine optimal PHCC tasking criteria in major trauma to best direct this resource.

On-scene times were longer than those reported from the same region in 2005, and now show differences between PHCC and conventional ambulance response [24], likely reflecting differences in case-mix, recording methods and evolutions in pre-hospital practice. Presence of PHCC and increasing geographic isolation is associated with prolonged on-scene time in major trauma, but accounts for a small proportion of the overall variance seen, and future work could consider level of service response when assessing the impact of pre-hospital on-scene times on outcomes.

Increasing workload in, and likelihood of, PHCC over the study period is an expected finding that aligns with service developments and extended hours.

### Strengths and limitations

This is the first work to examine the distribution of major trauma in the region using isochrone mapping to build a local model of time to major trauma centre, with greater potential to identify areas of isolation from care than other geospatial methods such as straight-line distance. We identify service-level considerations relating to geography and mechanism of injury for patients who may be currently underserved by pre-hospital critical care.

This work is limited by the lack of availability of outcome data, however future research including longitudinal follow-up could use the geographical modelling techniques described to assess association between geographic isolation from care and outcomes in major trauma and critical illness. Isochrones were not adjusted for emergency vehicle response speed exceeding the legal maximum, aeromedical transportation was not included in modelling, and the time thresholds specified assume immediate departure. Nevertheless, it is likely that the isochrones described adequately represent and separate varying degrees of isolation from major trauma centre. While GNAAS is the predominant operator of PHCC in the region studied, this work may not capture the entirety of advanced pre-hospital care delivered in the area. Data surrounding the attendance of neighbouring PHCC services under mutual aid arrangements, particularly from Yorkshire Air Ambulance and Yorkshire Ambulance Service were not available for analysis. Further limitations include in the presence of missing data, a common feature of pre-hospital datasets [25]. Cases where incident location was not available were not included in any analysis, representing a greater proportion of PHCC than conventional ambulance response, and pre-hospital deaths are not included in the reference database. Together, these factors mean that the work does not consider every case of major trauma in the region, and while missingness is a feature of pre-hospital registries, these limitations may limit the generalisability of the results [26]. Additionally, there is variation in the availability and operation of PHCC in England [27], and the service response demonstrated in this work may not be applicable to all regions here, or internationally. Future work may seek to explore national patterns of service provision and response to determine optimal operating models.

## Conclusion

This work has demonstrated increasing likelihood of pre-hospital critical care attendance with increasing geographic isolation from major trauma centre, alongside evidence of discrepancy of PHCC tasking in severe injury depending on mechanism. Advanced pre-hospital care may be of greater utility to those who require transportation over longer timeframes to major trauma centre, and the structure, dispatch and location of PHCC teams should seek to integrate these findings as services are conceived and develop.

### Electronic supplementary material

Below is the link to the electronic supplementary material.

Supplementary Material 1

## Data Availability

The datasets analysed during the current study are available from the corresponding author on reasonable request subject to appropriate data safeguards.

## List of abbreviations

GNAAS: Great North Air Ambulance Service
PHCC: Pre-Hospital Critical Care
