# Supplementary material for: The association between geospatial and temporal factors and pre-hospital response to major trauma: a retrospective cohort study in the North of England
Source: Scand J Trauma Resusc Emerg Med. 2023 Dec 19;31:103. doi: 10.1186/s13049-023-01166-x (PMC10729533; doi:10.1186/s13049-023-01166-x)
Supplement: Supplementary file 1 — Supplementary Material 1 [file 13049_2023_1166_MOESM1_ESM.docx]

**The association between geospatial and temporal factors and pre-hospital response to major trauma: A retrospective cohort study in the North of England.**

**Supplementary Appendix**

**Supplementary Figure 1.**

**Box Plots assessing distributions for linear regression model of on-scene time.**

**
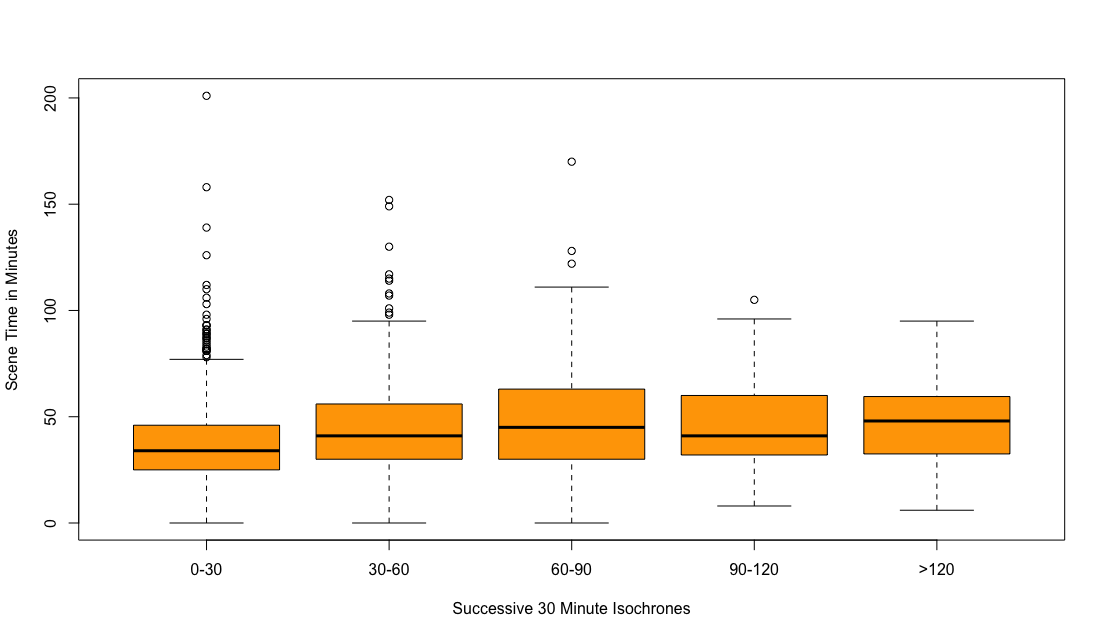
**

**
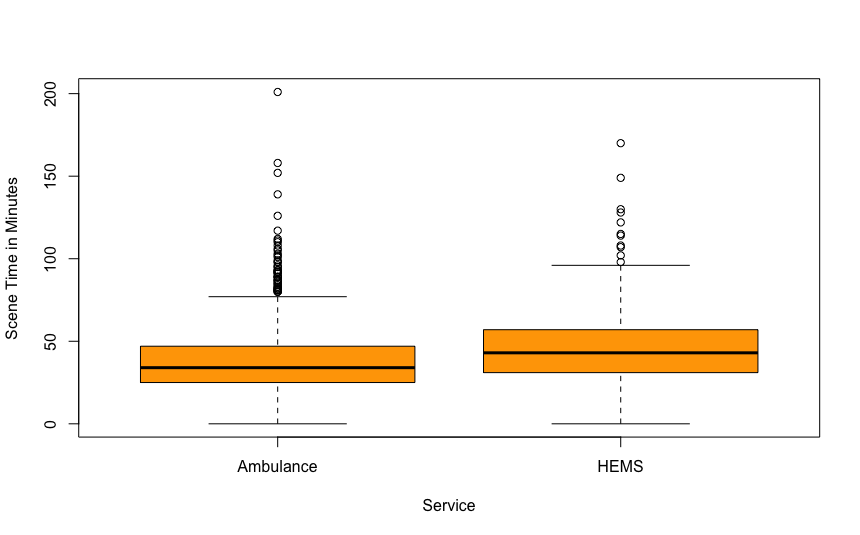
**

**Supplementary Table 1.**

**Results of post-hoc ordinal regression model of association between mechanism of injury and successive 30**

| **Univariate Ordinal Regression** | |
| --- | --- |
|  | **Odds Ratio of mechanism occurring in each successive 30-minute isochrone (95% Confidence Interval)** |
| **Mechanism** | |
| **Interpersonal Violence** | Reference |
| **Blast/Burn/Crush** | 1.01 (0.48-2.00) |
| **Fall of <2m** | 1.28 (0.97-1.71) |
| **Fall of >2m** | 1.34 (1.02-1.79) |
| **Vehicle Incident** | 1.61 (1.23-2.11) |
| **Other** | 0.63 (0.41-0.96) |
